# Effects of Terrestrial Buffer Zones on Amphibians on Golf Courses

**DOI:** 10.1371/journal.pone.0039590

**Published:** 2012-06-27

**Authors:** Holly J. Puglis, Michelle D. Boone

**Affiliations:** Department of Zoology, Miami University, Oxford, Ohio, United States of America; Monash University, Australia

## Abstract

A major cause of amphibian declines worldwide is habitat destruction or alteration. Public green spaces, such as golf courses and parks, could serve as safe havens to curb the effects of habitat loss if managed in ways to bolster local amphibian communities. We reared larval Blanchard's cricket frogs (*Acris blanchardi*) and green frogs (*Rana clamitans*) in golf course ponds with and without 1 m terrestrial buffer zones, and released marked cricket frog metamorphs at the golf course ponds they were reared in. Larval survival of both species was affected by the presence of a buffer zone, with increased survival for cricket frogs and decreased survival for green frogs when reared in ponds with buffer zones. No marked cricket frog juveniles were recovered at any golf course pond in the following year, suggesting that most animals died or migrated. In a separate study, we released cricket frogs in a terrestrial pen and allowed them to choose between mown and unmown grass. Cricket frogs had a greater probability of using unmown versus mown grass. Our results suggest that incorporating buffer zones around ponds can offer suitable habitat for some amphibian species and can improve the quality of the aquatic environment for some sensitive local amphibians.

## Introduction

Habitat loss is the number one cause of the biodiversity crisis [1]. Humans destroy or alter the landscape for residential, agricultural, commercial, and recreational use. Since 1945, it is estimated that urban land area in the United States has nearly quadrupled from about 15 million to 60 million acres [2]. If wildlife declines are to be curbed, conservation efforts will need to focus on protecting habitat as well as managing areas of human use in ways that minimize impact on wildlife.

At least 43% of known amphibian species are experiencing population declines [3] and are, therefore, in desperate need of conservation. Amphibians can act as indicators of ecosystem stress because amphibians are thought to be sensitive to changes in the environment since they utilize both aquatic and terrestrial habitat [4], but see Kerby et al. [5]. Amphibians play an important role in the ecosystems they inhabit. Amphibians are integral in their food webs by serving as both predator and prey. Through their diet amphibians can control populations of pest insects, such as mosquitoes [6] and algae [7]. Amphibians can also assimilate a large amount of the energy they ingest [6]–[10] and convert their food resources to biomass, which serves as a prey base for higher trophic levels. Studies have indicated that amphibian biomass can exceed that of other taxonomic groups such as birds and small mammals [11] and that removal of amphibians can depress plant production and alter nutrient cycling [10], [12]. By conserving amphibian populations, the services they provide in their ecosystems will also be preserved. Because the leading cause of amphibian declines worldwide is habitat destruction and alteration [3], managed green spaces offer an opportunity for suitable amphibian habitat to maintain these services in altered habitats if we understand how management practices can affect native species.

Recreationally managed green spaces such as parks and golf courses may partly mitigate the effects of habitat loss. There are more than 17,000 golf courses in the United States comprising over half a million hectares of land [13]. On a golf course, up to 70% of the course is considered “rough” or out of play [14], leaving a large area of land that if managed in ways consistent with natural habitats, could provide habitat to some native species of wildlife. In fact, Colding and Folke [14] found golf courses to have a higher ecological value in many cases than other land types, such as parkland, agricultural, residential, and highly urban lands based on measurements of species diversity, richness, abundance, and other measures of biota. Golf courses could benefit from diverse populations of animals, such as amphibians, because amphibians could reduce the cost of managing the course through providing valuable services. As larvae, tadpoles eat algae and salamanders eat aquatic invertebrates, and as juveniles and adults they can eat insects, such as mosquitoes. This may reduce the need to stock fish in ponds, use algaecides/herbicides to control algal growth, or spray pesticides to manage mosquitoes creating a win-win situation for managers and wildlife.

Golf courses often contain aquatic habitat, such as ponds or wetlands, which is integral to amphibians with complex life cycles. Previous research has shown that amphibians will use golf course ponds, but that most courses have lower amphibian biodiversity than reference sites because of a lack of hydroperiod variability [15]. Boone et al. [16] also found that amphibian survival on golf courses in Missouri equaled or exceeded survival on reference sites in some cases, implicating lower macroinvertebrate predator abundance in golf course ponds as one reason for higher survival. These studies suggest that some amphibians can utilize golf courses for a portion of the life cycle, but do not address whether golf courses provide the necessary terrestrial habitat for the completion of amphibian life cycles, which is essential for population persistence.

Suitable adjacent or upland terrestrial habitat is required for amphibians with complex life cycles [17]; however, terrestrial life stages are less studied in natural, as well as managed, habitats. Golf courses have green space, but the quality is likely compromised by physical alteration, such as mowing or soil compaction, and by chemical management with pesticides and nutrient supplementation, such as nitrogen and phosphorus fertilizers [18]. The effects of some of these management practices can potentially be minimized with the presence of terrestrial buffer zones.

Taller, unmown grass around ponds may provide essential habitat for juvenile and adult amphibians because it may harbor more insects, thus more food, suitable overwintering sites, and protection from desiccation and predators. If juvenile and adult amphibians use this habitat, it could increase the probability of survival and slow population declines, but more research is needed to understand the effect of changes in the terrestrial environment on amphibians. In addition to providing habitat for juvenile and adult amphibians, terrestrial buffer zones could also reduce contaminants such as pesticide, nutrient, and sediment loads from runoff [19]–[21]. Terrestrial buffer zones filter sediment-bound nutrients and pesticides by slowing the velocity of the runoff to allow for deposition and for soluble materials to be adsorbed into plants and soil [21]–[23]. The United States Department of Agriculture's Natural Resources Conservation Service already considers buffer zones a best management practice for reducing nonpoint source pollution [24].

To investigate whether unmown, terrestrial buffer zones around golf course ponds can be used to support aquatic and terrestrial life stages of amphibians, we used two anurans, Blanchard's cricket frog (*Acris blanchardi*) and green frogs (*Rana clamitans*). Blanchard's cricket frog is a widespread grassland species experiencing enigmatic population declines in parts of its range [25]–[28] and is associated with permanent water bodies [29], the most common type of aquatic habitat created by humans for recreation or aesthetics. Both juvenile and adult cricket frogs utilize the perimeter of ponds [30], which makes this species an ideal candidate for examining the effects of a terrestrial buffer zone. Green frogs are also widespread and are considered habitat generalists. These frogs are commonly associated with human dominated landscapes such as mitigation ponds, parks, and golf courses [15], [31]–[32]. However, because green frogs overwinter as larvae, we only used green frog tadpoles in the larval study and not in any of the terrestrial experiments.

We examined the effects of terrestrial buffer zones on the full life cycle of amphibians by conducting a series of studies. First, we assessed the effects of buffer zones on larval survival and development using both cricket frogs and green frogs reared separately in enclosures within golf course ponds with and without terrestrial buffer zones surrounding the pond. Second, to examine the effects of buffer zones on the terrestrial phase of the life cycle, we followed Blanchard's cricket frogs reared in golf course ponds with and without buffers through overwintering with a mark-recapture study. Third, we assessed juvenile cricket frog preference for mown versus unmown grass in enclosure experiments.

Due to the filtering nature of the terrestrial buffer zones, we expected greater survival and mass for both larval green frogs and metamorphosed cricket frogs reared in ponds with buffer zones than those reared in ponds without buffer zones because buffer zones should reduce mortality from direct toxicity of contaminants to amphibians. In the terrestrial environment, we predicted juvenile cricket frogs would have a greater survival and affinity for unmown grass (or buffer zones) over mown grass (or unbuffered zones) when given a choice because of the potential positive effects of greater food abundance and lower desiccation in unmown grass. The main objectives of this research were to evaluate how common practices on managed areas can affect amphibians and to develop simple management strategies that can be implemented to improve the possibility that amphibians could have sustainable populations on golf courses and other managed green spaces.

## Methods

### Ethics Statement

The experiments described here comply with current laws of the U.S. and the state of Ohio. The research was approved and conducted under animal care protocol 740 by the Institutional Animal Care and Use Committee at Miami University. Animals were collected according to Ohio Department of Natural Resources regulations under collectors permit #11–87.

### Effects of Buffer Zones on the Aquatic and Terrestrial Life Stages of Amphibians on Golf Courses

We collected 16 pairs of Blanchard's cricket frogs in amplexus between 5-May-2008 and 14-June-2008 and 18 pairs between 25-June-2008 and 16-July-2008 from an uncontaminated pond at Miami University's Ecology Research Center in Oxford, Ohio (U.S.A.). Each pair was kept in a 2 L plastic container with 5 centimeters of pond water overnight. The adults were returned to the pond the following morning after laying eggs. We collected three egg masses of green frogs from a forested pond in Miami University's Natural Areas in Oxford, Ohio (U.S.A.). We kept Blanchard's cricket frog and green frog eggs in the laboratory until use in the study. After hatching, cricket frog tadpoles from the first 16 clutches were combined as were tadpoles from the last 18 clutches. All three green frog clutches were also combined. Tadpoles were fed TetraMin fish flakes ad libitum and water was changed daily until added to the field enclosures.

We placed tadpoles in enclosures at three local golf courses: Hueston Woods Golf Course (College Corner, Ohio, Butler County), Oxford Country Club Golf Course (Oxford, Ohio, Butler County) and Twin Run Golf Course (Hamilton, Ohio, Butler County). Hueston Woods Golf Course is a public golf course associated with a state park. The staff used a more naturalistic approach in the management of the golf course, compared to the two other courses in this study, and was already leaving large tracts of land unmown on the course. Both ponds on this course are permanent ponds that do not dry out. Twin Run Golf Course is also a public course and appeared to have the most active chemical management. In fact, the buffered pond used at this location was dosed with copper sulfate during the course of our study because of concern over excessive aquatic vegetation by the golfers and managers. Again, both ponds on this course are permanent ponds that never dry out throughout the year. Finally, Oxford Country Club is a privately managed course and in general, staff used the ponds on the course mostly for irrigation and thus the water levels fluctuated regularly in the ponds. The ponds on this course have variable hydroperiods and have completely dried out on occasion. At each golf course, we used two ponds, one with an approximately 1 m grass buffer zone and one without. To create the buffer zone, golf course staff did not mow 1 m from the pond and allowed the grass to grow taller while ponds without a buffer zone were mowed all the way to the ponds edge.

On 1-July-2008, we added five green frog field enclosures to each of the ponds and on 22-July-2008, we added five Blanchard's cricket frog field enclosures to each of the ponds. Enclosures were cylindrical and made of fiberglass screening (with a 1 mm×2 mm mesh size). They were approximately 0.5×2 m and we placed a 1×3 m piece of hardware netting (approximately 4 cm×4 cm mesh size) inside the enclosure to maintain the cylindrical structure. One meter from the bottom of the enclosure, we attached two flotation devices (i.e., pool noodles). Each enclosure was secured to a post in the water and the tops were rolled down and pinned to the posts with binder clips. We added 0.5 kg of deciduous leaf litter for refuge to each enclosure the same day they were placed in the ponds.

On 2-July-2008, we haphazardly added 40 green frog tadpoles from combined clutches to each enclosure. Blanchard's cricket frog tadpoles were added on two days because we were not able to collect enough individuals at once and to minimize differences in amount of time that tadpoles spent in the lab. Therefore, on 23-July-2008, we haphazardly added 20 Blanchard's cricket frog tadpoles from combined clutches to each of the cricket frog field enclosures and on 4-Aug-2008 we haphazardly added another 20 cricket frog tadpoles to those same enclosures for a total of 40 tadpoles in each enclosure. This is within the range of natural densities for larval amphibians [33]_._

We monitored enclosures daily for metamorphosed amphibians. We collected the metamorphs with a net and placed all individuals from the same enclosures into a plastic container with some pond water. Metamorphs were brought to the lab and we recorded their mass and time to metamorphosis. Each metamorph was given a permanent mark by toe-clipping, but no more than one toe on each foot. Toe clipping is a widely used method of marking amphibians [34]–[36]. We returned metamorphs on the following day to terrestrial habitat surrounding the golf course pond where they were reared in enclosures. Only Blanchard's cricket frogs metamorphosed because green frogs typically overwinter in the pond as tadpoles and do not emerge as metamorphs until the following year. On 9-Oct-2008, we removed all enclosures from the pond and collected surviving tadpoles. Tadpoles were brought back to the lab and we weighed and developmentally staged [37] each tadpole. We returned the green frog tadpoles to their natal pond.

On 9-July, 23-July, 20-Aug, and 4-Sept-2008 we collected water samples from all green frog enclosures in each pond and on 30-July, 14-Aug, and 4 Sept-2008 we collected water samples from all Blanchard's cricket frog enclosures in each pond. From each sample, we took 100 ml of water and filtered it onto glass filter paper. The filters were placed in buffered acetone and refrigerated for 24 hours. We then analyzed the sample chlorophyll a by fluorometry to estimate relative phytoplankton abundance. On these collection dates, we also measured temperature, pH, and dissolved oxygen (DO) in each pond.

On 18 Sept-2008, we sampled macroinvertebrates by doing three to six 2 m sweeps in each pond outside of the enclosures with a dip net. If anything was collected in the first three sweeps, we only sampled three times. However, if nothing was collected in the first three sweeps, we sampled an additional three times. Individuals were identified to taxonomic group in the field and released. This was a cursory survey and we did not collect enough data for a quantitative assessment of invertebrate predator differences among ponds, however all of the ponds were similar in the composition and density of macroinvertebrates.

For Blanchard's cricket frogs, we determined days to metamorphosis, mass at metamorphosis, and percent survival to metamorphosis. The percent survival to metamorphosis was the same as total survival for the cricket frogs as all surviving tadpoles had reached metamorphosis when we terminated the experiment. For green frogs, we calculated percent tadpole survival, mass, and developmental stage [37] at the termination of the study. Survival data was angularly transformed, mass was log transformed, and developmental stage and days to metamorphosis were rank transformed prior to analysis. The data met the assumptions of analysis of variance (ANOVA) for all variables except days to metamorphosis for cricket frogs and Gosner developmental stage for green frogs; these variables were rank transformed, which resolves issues in normality for ANOVA.

To test for the effects of buffer zone treatment and golf course block, amphibian responses were analyzed with one-way nested ANOVA with two error terms. To test for differences among golf courses we used the nested term buffer nested within golf course (i.e., Buffer [Golf]) as the error term. To test for differences between buffered and unbuffered ponds, we tested the buffer nested within golf course term (Buffer [Golf]) against the residual error, enclosure nested within buffer zone (i.e., Encl [Buffer]), to take into account that enclosures were nested within ponds to avoid pseudoreplication. Survival was used as a covariate in the analyses for mass and time to metamorphosis for cricket frogs and for stage and mass at the end of the study for green frog tadpoles. In cricket frog analyses, the Oxford Country Club golf course site was eliminated from analyses because some animal(s) chewed large holes in the enclosures and the tadpoles escaped.

Phytoplankton effects were tested with nested ANOVAs. To test for differences in phytoplankton abundance over time, the main effect of time was tested against the residual error (Time * Encl). Enclosures within pond (Encl [Buffer*Golf]) were tested against residual error, as were differences among golf courses over time (Golf * Time), and differences between buffered and unbuffered ponds among ponds over time (Buffer * Time [Golf]). The buffered effect was tested against enclosures within ponds (Encl [Buffer*Golf]). Phytoplankton was angularly transformed prior to analysis. All data met the assumptions of ANOVA.

DO, pH, and temperature were also analyzed with nested ANOVAs. To test for differences among golf courses, the effect of golf course (Golf) was tested against pond (Buffer [Golf]). The buffer effect (Buffer [Golf]), or pond, was tested against the residual error, as were the effects of time and the interaction of the golf course effect over time. All data met the assumptions of ANOVA.

At Twin Run, 41 juveniles were released at the unbuffered pond and 17 were released at the buffered pond. At Hueston Woods Golf Course, 109 juveniles were released at the buffered pond and 48 were released at the unbuffered pond. At Oxford Country Club, 11 juveniles were released at both ponds. Eleven of the frogs released at each site were reared in mesocosms from a separate study (see [38] for details); the remaining frogs were survivors from the larval study conducted on the golf courses in the summer of 2008. The number of cricket frogs released reflected the number collected from the enclosures at a given pond; so the number released varied at each pond. We gave each juvenile a unique toe clip code to identify them upon recapture. We returned the following spring and summer to each golf course pond to locate adult Blanchard's cricket frog survivors. Each pond was visited at least three times in the early morning (between 6 and 7:00 a.m.). At this time, cricket frogs were calling at a reference site and some of the golf course ponds, and we routinely found cricket frogs at these sites. We collected animals at some of the sites; however, none of the animals collected had any identifying toe clips. We were unable to locate any survivors from the enclosure experiment from the previous summer.

### Blanchard's Cricket Frog Choice Between Mown and Unmown Habitat in Terrestrial Pens

On 23-July-2009, we collected 80 recently metamorphosed juvenile Blanchard's cricket frogs from a pond on Miami University's Ecology Research Center in Oxford, Ohio. We brought all the animals into the lab and held them in individual containers until tail resorption. After tail resorption, we obtained the animals' mass and it was given an identifying toe clip by clipping no more than one toe on each foot. We held all animals in the lab and fed them small crickets ad libitum until the start of the study.

We constructed eight pens that were 3 m×3 m with silt fencing that once buried, were approximately 1 m tall, in a grassy field at Miami University's Ecology Research Center. In each pen, we randomly mowed the grass in half of the pen and left the other half unmown. On 27-July-2009, we haphazardly assigned ten frogs to each of the eight pens and released them into the pens at 10:00 p.m. At that time, we also measured the soil moisture, relative humidity and temperature in each treatment, mown and unmown grass, in each pen. On 31-July-2009 at 1:00 p.m., we placed silt fence barriers between the mown and unmown grass in each pen. We collected the juvenile cricket frogs from each section and placed them in containers with damp paper towels together with all frogs caught from the same section in the same pen. We returned the animals to the lab and recorded their mass after which they were released to the pond where we collected them.

On 28-July-2009 we collected insects by sweep netting in a mown and unmown area adjacent to the pens. We sampled three unmown transects and three mown transects with ten sweeps for each transect. After completing each transect, we collected insects in a large plastic Ziploc bag and placed each bag in the freezer. Each replicate was bagged separately. Insects were weighed to determine potential food differences between mown and unmown habitat.

We determined that a cricket frog made a choice of one habitat over the other (mown vs. unmown grass) if it was located on one side of the barrier vs. the other. To test for the effects of cricket frog habitat on choice we analyzed the data with a Hotelling's T-squared test using the proportion of individuals that were found on each side. We analyzed relative humidity, temperature, soil moisture, and insect biomass data with Hotelling's T-squared test to test for differences between mown and unmown grass.

We conducted an additional study to determine if the likelihood of detection of cricket frogs was influenced by habitat. On 6-May-2010 we collected 40 adult male cricket frogs from a pond on Miami University's Ecology Research Center. The animals were brought back to the lab, weighed, and given identifying toe clips by clipping no more than one toe on each foot. Animals were held overnight and fed crickets ad libitum. We used the same pens as the juvenile cricket frog choice study from the previous year. The pens were prepared the same way as the previous summer with one half of each pen randomly mowed and the other half was left unmown. We placed the barrier between the two sides of each pen prior to adding animals and released five adult male cricket frogs on each half of each pen on 7-May-2010. The following day we returned to the pens to locate animals. We spent approximately five minutes in each section of all eight pens. When we located an animal, it was identified and placed him in a container with moist paper towels until our search time expired and then all animals located in that section were released back in the pen. This same process was repeated daily for the following two days. To determine whether there was a bias in locating animals in the mown or unmown grass, we analyzed the average number of animals found in mown and unmown grass over the three days with a one-way ANOVA. We did not use Hotelling's T-squared test because the number of animals recovered in mown and unmown grass are not interdependent in this design. All data met the assumptions of ANOVA.

## Results

### Effects of Buffers on the Aquatic and Terrestrial Life Stages of Amphibians on Golf Courses

#### Green Frogs

**Figure 1:**
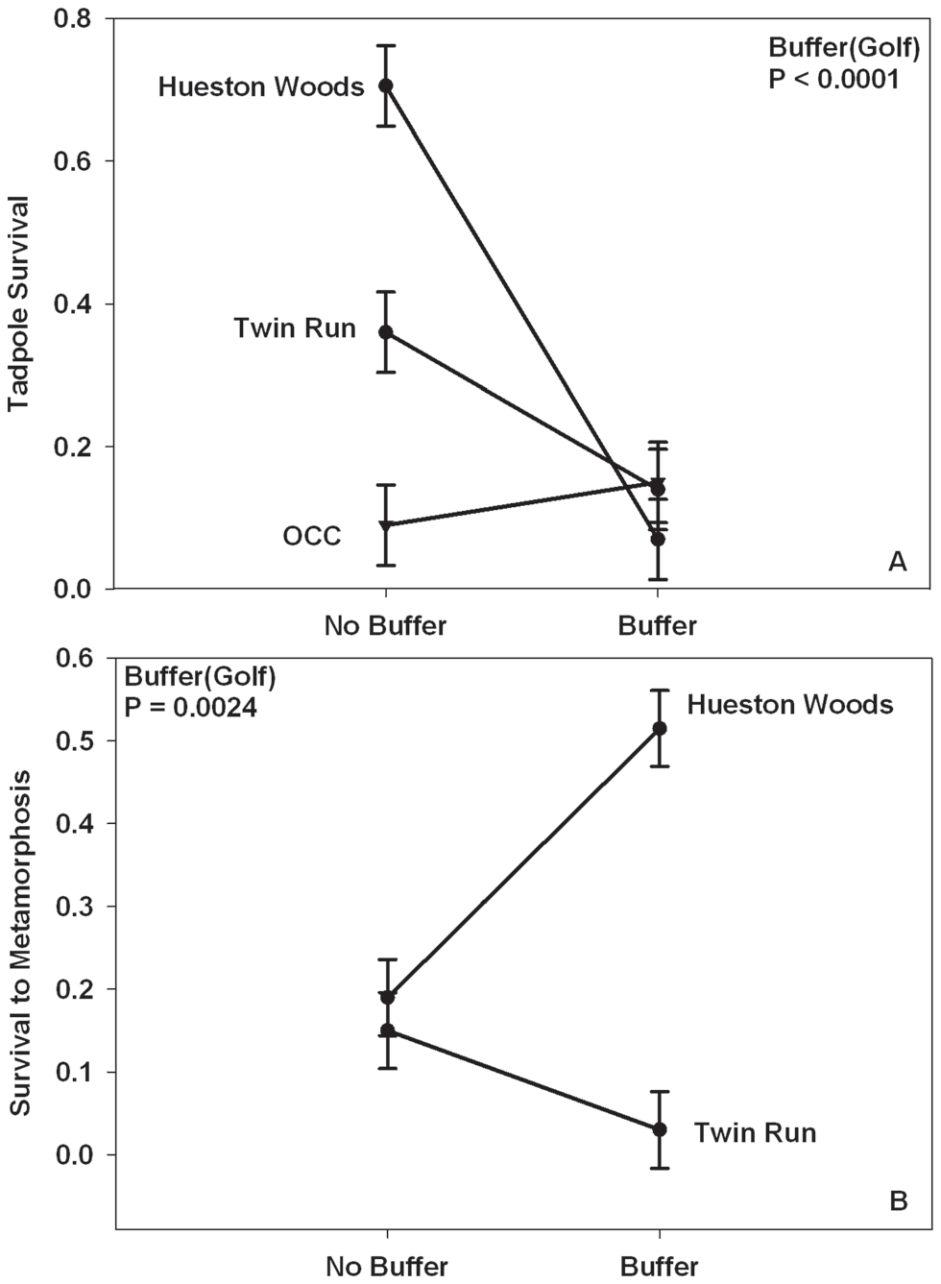
Survival of green frogs and cricket frogs in buffered and unbuffered golf course ponds. Shown is survival of (A) green frog tadpoles to end of study and (B) cricket frog tadpoles to metamorphosis reared in buffered and unbuffered ponds on golf courses (OCC =  Oxford Country Club). Error bars represent one standard error of the mean.

**Figure 2:**
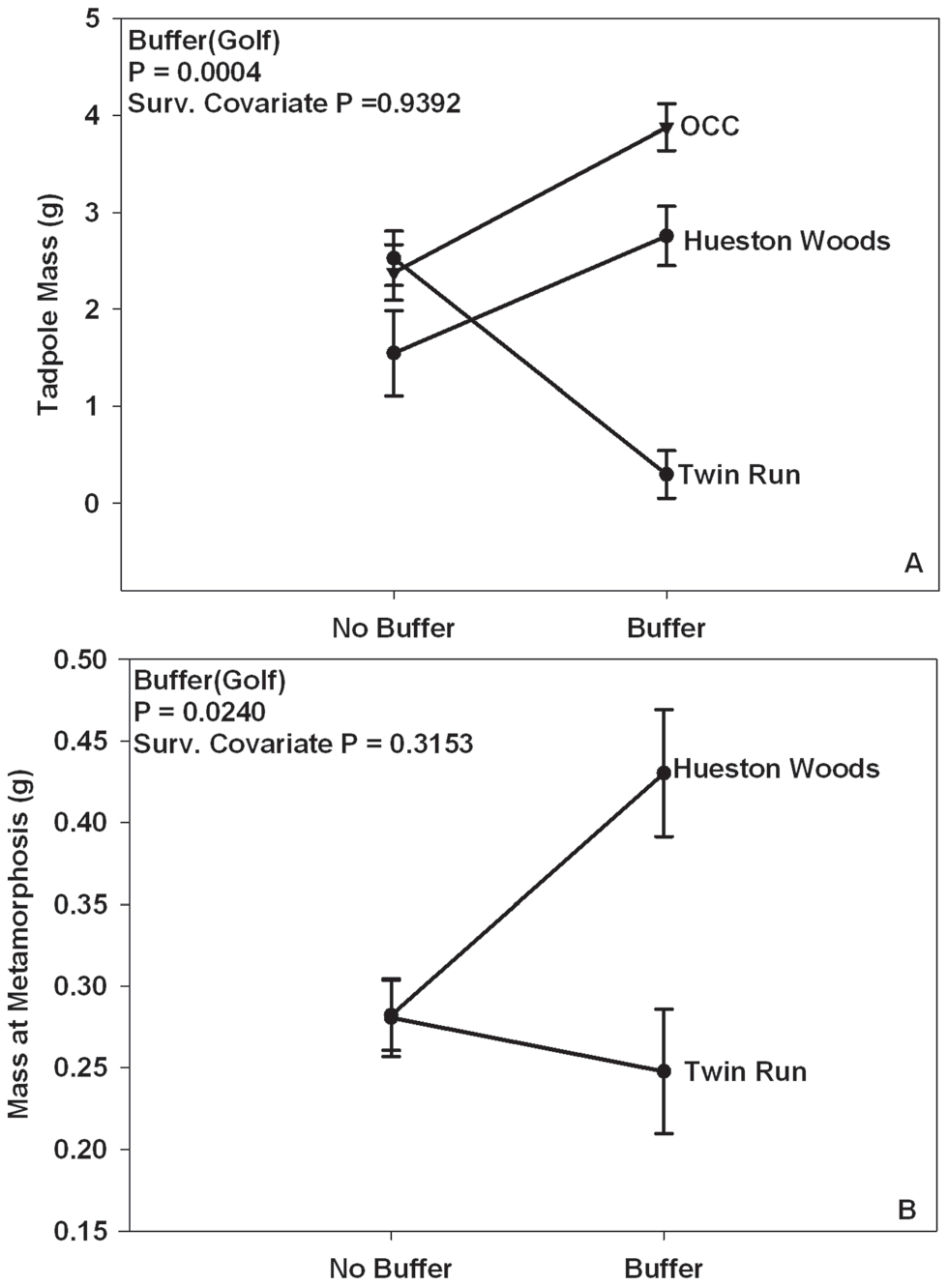
Mass of green frogs and cricket frogs in buffered and unbuffered golf course ponds. Shown is mass of (A) green frog tadpoles at end of study (B) cricket frog mass at metamorphosis reared in buffered and unbuffered ponds on golf courses (OCC =  Oxford Country Club). Error bars represent one standard error of the mean.

There were significant differences in survival between buffered and unbuffered ponds among golf courses (Buffer [Golf]), with green frog tadpoles reared in buffered ponds on Hueston Woods and Twin Run having lower survival than those tadpoles reared in unbuffered ponds (Table 1; Fig. 1A). Also, green frog tadpole mass was significantly different between buffered and unbuffered ponds among golf courses with those animals reared in buffered ponds on Hueston Woods and Oxford Country Club having a greater mass at the end of the study than those tadpoles reared in unbuffered ponds on those courses (Table 1; Fig 2A). In contrast, green frogs at Twin Run reared in the buffered pond had a smaller mass than those animals reared in the unbuffered pond (Table 1; Fig. 2A). However, there were no significant differences in green frog tadpole survival, tadpole mass, or tadpole developmental stage at the end of the study among animals reared in different golf courses (Table 1). Also, there were no differences in developmental stage at the end of the study between buffered and unbuffered ponds (Table 1).

**Table 1:** Summary of nested analyses of variance (ANOVAs) for green frog survival, mass, and developmental stage at the end of the study and Blanchard's cricket frog survival to, mass at, and days to metamorphosis.

Response Variable	Treatment	*df*	Mean Square	*F*	*p*
Green Frog					
Tadpole Survival	Golf	2,3	0.2695	0.52	0.6386
	Buffer(Golf)	3,24	0.5155	11.39	<0.0001
Tadpole Mass	Covariate (Surv)	1	0.0001	0.01	0.9392
	Golf	2,3	0.1848	0.69	0.5661
	Buffer (Golf)	3,19	0.2670	9.96	0.0004
Gosner Stage	Covariate (Surv)	1	34.89	0.01	0.9081
	Golf	2,3	3006	0.38	0.7105
	Buffer (Golf)	3,19	7830	3.07	0.0527
Blanchard's Cricket Frog					
Survival to Metamorphosis	Golf	1,2	0.6689	3.04	0.2232
	Buffer(Golf)	2,16	0.2197	9.02	0.0024
Mass at Metamorphosis	Covariate (Surv)	1	0.0231	1.09	0.3153
	Golf	1,2	0.1238	1.16	0.3940
	Buffer(Golf)	2,13	0.1067	5.03	0.0240
Days to Metamorphosis	Covariate (Surv)	1	7.146	0.01	0.9299
	Golf	1,2	55.76	0.02	0.8964
	Buffer(Golf)	2,13	2567	2.89	0.0915

The covariate in the mass and stage/days to metamorphosis analyses is tadpole survival (Surv) for green frogs and survival (Surv) to metamorphosis for cricket frogs.

#### Blanchard's Cricket Frogs

There were significant differences in survival to and mass at metamorphosis between cricket frogs reared in buffered and unbuffered ponds between golf courses (Table 1). Cricket frogs on Hueston Woods had greater survival (Fig. 1B) and were larger (Fig. 2B) when reared in buffered ponds than when reared in unbuffered ponds. In contrast, there were no differences in survival (Fig. 1B) or mass at metamorphosis (Fig. 2B) between frogs reared in buffered and unbuffered ponds on Twin Run Golf Course. Also, there were no significant differences in Blanchard's cricket frog survival to, mass at, or days to metamorphosis (Table 1) among animals reared in different golf courses. There were also no significant differences in cricket frog days to metamorphosis between animals reared in buffered and unbuffered ponds (Table 1). Oxford Country Club was left out of these analyses because some animal(s) chewed large holes in the enclosures in these ponds and the animals escaped.

We were unable to locate any adult Blanchard's cricket frog survivors on the golf courses in the summer of 2009. Therefore, we were unable to determine if the buffer zone had any effect on adult survival or reproduction.

#### Water Quality

**Figure 3:**
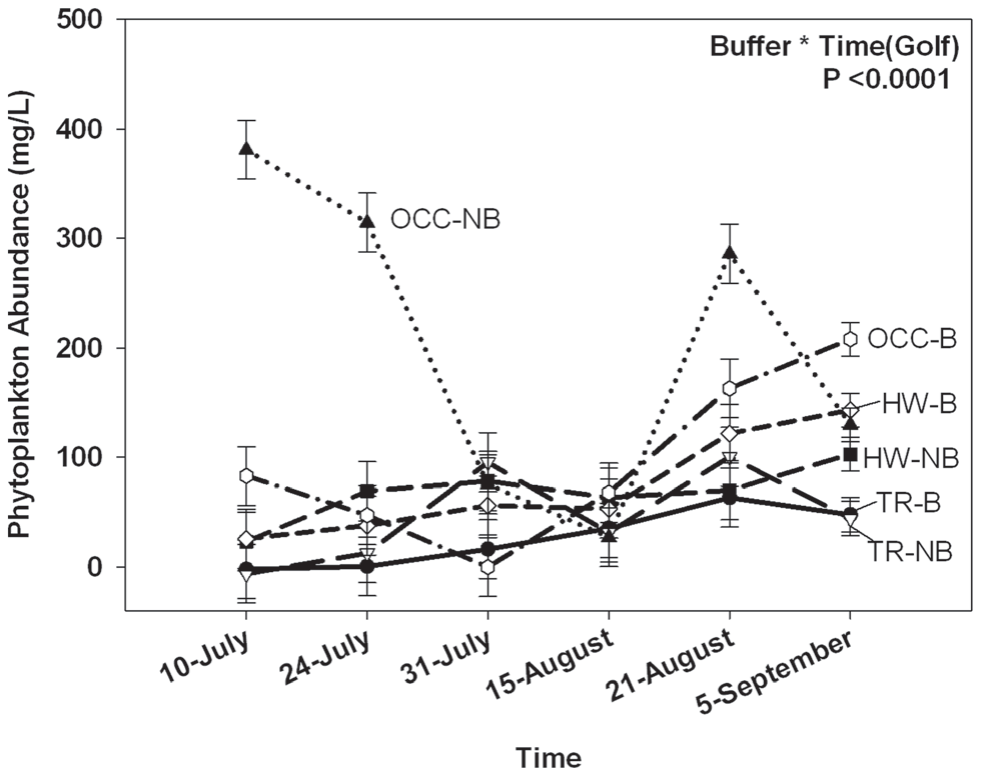
Changes in the abundance of phytoplankton in buffered and unbuffered golf course ponds over time. Shown is phytoplankton abundance (µg/L) measured in buffered and unbuffered golf course ponds from July-Sept-2008. HW =  Hueston Woods, TR =  Twin Run, OCC =  Oxford Country Club, NB =  No Buffer Zone, and B =  Buffer Zone. Error bars represent ± 1 SE.

There were significant differences in phytoplankton abundance among golf courses with Oxford Country Club having the greatest abundance and Twin Run having the least (Table 2), between buffered and unbuffered ponds among golf courses (Table 2, Table 3), and also over time (Table 2). There were also significant interactions with golf course and time (Table 2), and between buffered and unbuffered ponds among golf courses over time (Table 2, Fig. 3). In general the buffered pond at Hueston Woods had greater phytoplankton abundance than the unbuffered pond, while the unbuffered pond at Oxford Country Club had greater phytoplankton abundance than the buffered pond. Also, phytoplankton abundance generally increased over time (Fig. 3). Phytoplankton abundance was not significantly different among enclosures within ponds (Table 2).

**Table 2:** Summary of nested ANOVAs for phytoplankton abundance, pH, dissolved oxygen (DO), and temperature.

Response Variable	Treatment	*df*	Mean Square	*F*	*p*
Phytoplankton	Golf	2,3	45.6006	9.76	0.0486
	Buffer(Golf)	3,54	4.6702	13.25	<0.0001
	Time	5,120	9.5724	32.37	<0.0001
	Golf*Time	10,120	1.9960	6.75	<0.0001
	Buffer*Timec(Golf)	15,120	2.2714	7.68	<0.0001
pH	Golf	2,3	1.5071	1.90	0.2933
	Buffer(Golf)	3,15	0.7940	4.18	0.0244
	Time	5,15	3.1119	16.39	<0.0001
	Time*Golf	10,15	0.3461	1.82	0.1420
DO	Golf	2,3	29.4501	1.20	0.4133
	Buffer(Golf)	3,15	24.4757	5.22	0.0115
	Time	5,15	4.2306	0.90	0.5050
	Time*Golf	10,15	14.0507	3.00	0.0272
Temperature	Golf	2,3	3.1477	0.39	0.7089
	Buffer(Golf)	3,15	8.1388	10.95	0.0005
	Time	5,15	13.0024	17.49	<0.0001
	Time*Golf	10,15	1.9751	2.66	0.0428

**Table 3:** Least squares means [± 1 SE] for phytoplankton, pH, DO, and temperature of buffered and unbuffered ponds over time on each golf course.

	Twin Run	Hueston Woods	Oxford Country Club
Phytoplankton (μg/L)			
Unbuffered	26.6690[8.9641]	67.6400[8.9641]	202.0800[8.9641]
Buffered	46.1846[8.9641]	72.7733[8.9641]	94.6566[8.9641]
DO (mg/L)			
Unbuffered	6.7383[0.8841]	6.6783[0.8841]	5.6166[0.8841]
Buffered	3.6000[0.8841]	9.9216[0.8841]	7.6433[0.8841]
pH			
Unbuffered	8.1716[0.1778]	8.2583[0.1778]	7.8550[0.1778]
Buffered	7.6383[0.1778]	8.8833[0.1778]	8.2000[0.1778]
Temperature (°C)			
Unbuffered	26.7500[0.3520]	26.4000[0.3520]	25.2666[0.3520]
Buffered	25.0166[0.3520]	27.4000[0.3520]	27.3000[0.3520]

Phytoplankton was the only response with significant differences among golf courses, while the buffer treatment was significant for all responses below (Table 2)'.

There were significant differences in DO between buffered and unbuffered ponds among golf courses with the unbuffered pond on Twin Run having higher DO than the buffered pond and the opposite effect between the ponds at Hueston Woods and Oxford Country Club (Table 2, Table 3). There were also significant differences among golf courses over time with DO generally decreasing and then increasing over time at Twin Run, increasing over time at Hueston Woods, and decreasing over time at Oxford Country Club.

There were significant differences in pH over time (Table 2) with pH increasing between the first two sampling dates and then holding steady, and also between buffered and unbuffered ponds among golf courses with the unbuffered pond at Twin Run having a higher pH than the buffered pond and the opposite effect between ponds at both Hueston Woods and Oxford Country Club (Table 2, Table 3).

There were significant differences in temperature over time with it fluctuating between 25–28.1°C as well as among golf courses over time (Table 2). There were also significant differences between buffered and unbuffered ponds among golf courses with the unbuffered pond at Twin Run having a higher temperature than the buffered pond and the opposite effect between ponds at Hueston Woods and Oxford Country Club (Table 2, Table 3).

### Habitat Choice in Terrestrial Pens

When given a choice between mown and unmown habitat in terrestrial pens, a marginally greater proportion of juvenile cricket frogs chose unmown habitat (Table 4; Fig. 3). Relative humidity was significantly greater in unmown grass (55.8%+/− 0.901) than in mown grass (49.712%+/− 1.44) (Table 4). There was significantly lower insect biomass (Table 4) in mown (0.1344 g+/−0.0264) compared to unmown grass (0.4180 g+/−0.0606). Soil moisture did not differ significantly between mown and unmown grass (Table 4).

**Table 4:** Summary of Hotelling's T-squared test for Blanchard's cricket frog choice, and one-way ANOVAs for differences in relative humidity, soil moisture, insect biomass, and detection probability between mown and unmown grass.

Response Variable	*df*	Wilks' λ	Mean Square	*F*	*p*
Choice	1, 7	0.5629		5.49	0.0525
Relative Humidity	1, 7	0.2376		22.46	0.0021
Soil Moisture	1, 7	0.9635		0.26	0.6228
Insect Mass	1, 2	0.0850		21.52	0.0435
Detection	1, 12		9.1746	15.91	0.0018

**Figure 4:**
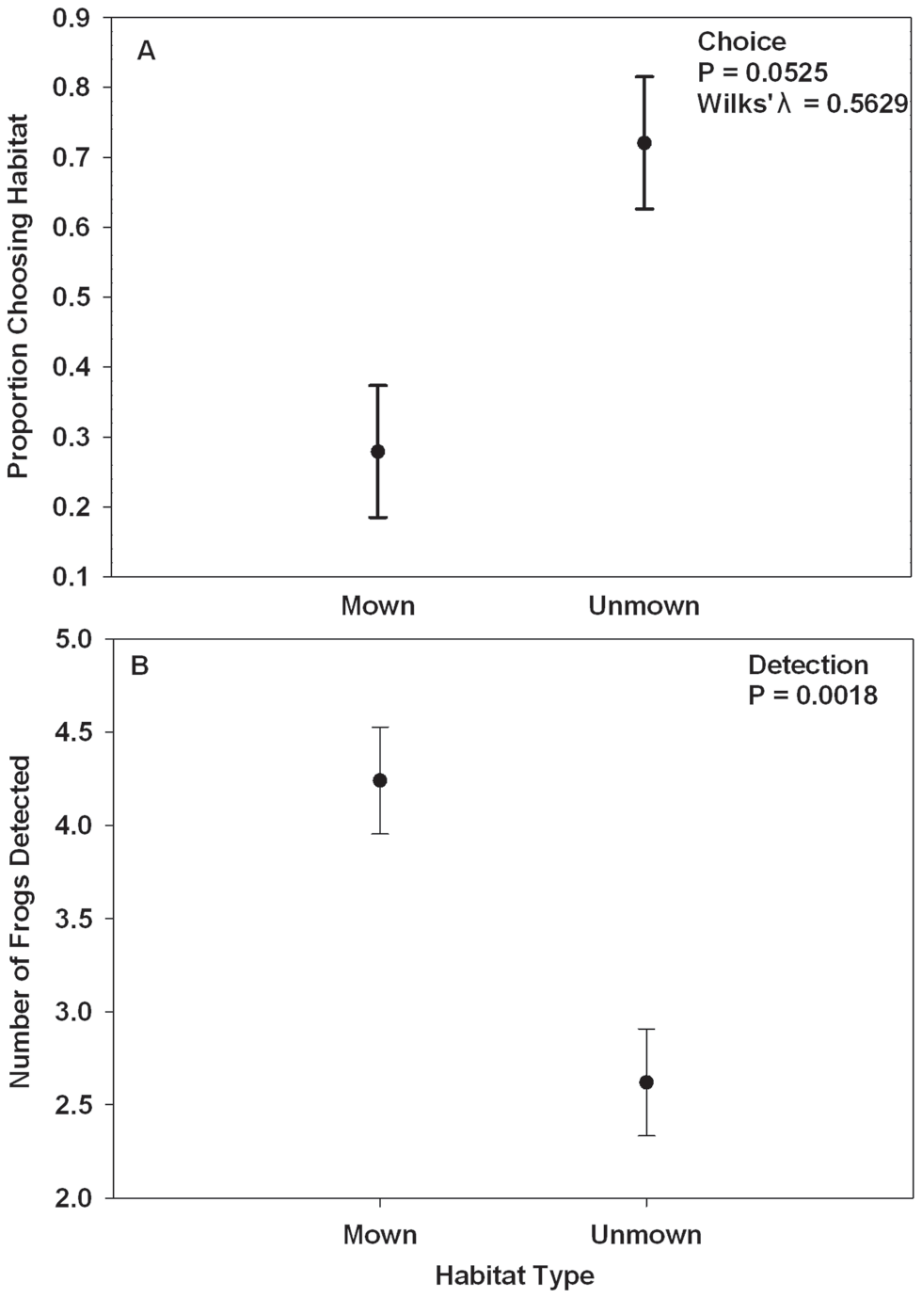
Habitat choice and detection rates in mown and unmown grass. Shown is (A) the proportion of juvenile Blanchard's cricket frogs choosing either mown or unmown habitat when given a choice between the two and (B) number of adult Blanchard's cricket frogs found, out of five animals, in mown and unmown grass. Error bars represent ±1 SE.

In another study, we placed cricket frogs in pens with mown or unmown grass so that we could determine if habitats differed in detection rates. We were able to locate adult cricket frogs in mown grass (4.2381+/−0.2870) significantly more than in unmown grass (2.6190+/−0.2870) (Table 4, Fig. 4b), suggesting a bias toward relocating cricket frogs in unmown grass.

## Discussion

Our research demonstrated the addition of buffer zones around ponds on managed green spaces, such as golf courses, can affect amphibian populations. Without altering management strategies, golf courses are more suited to common species such as green frogs and bullfrogs [15]. But our study indicates that with the addition of buffer zones, other species that may be more sensitive to environmental degradation, like the cricket frog, may find suitable habitat on golf courses.

### Impact of Buffer Zone on Aquatic Life Stage

Green frogs are commonly found in landscapes dominated by human activity [15], [31]–[32] and are potentially less sensitive to contaminants than other amphibian species [38]–[40]; therefore, green frogs may have experienced greater survival in unbuffered ponds because they have an advantage in a contaminated system. Ade et al. [38] found cricket frogs to be more sensitive to both the insecticide imidacloprid and aquatic predators than green frog larvae; therefore, increased survival of cricket frogs in the buffered ponds could indicate lower contaminant levels than in the unbuffered ponds, which could be expected because vegetation has been shown to filter out contaminants [21]–[23]. Regardless of the different effects of the buffer zone on the survival of green frogs and cricket frogs, in natural ponds, survival to metamorphosis is typically 2–5% [41]–[44]. Larval green frog survival and cricket frog survival to metamorphosis in our study was almost always well above this range (3–72%; Fig. 1A, B), which supports the findings of Boone et al. [16] that amphibians can complete larval development in golf course ponds in Missouri with equal or greater survival to those reared in more natural ponds, and suggests that changes in the terrestrial environment can have effects on the larval stage.

We expected and found some differences between golf course sites because each site is managed differently with varying types and levels of pesticides and fertilizers, mowing regimes, and pond uses. Some ponds were used simply as water hazards, an obstacle the golfers must avoid, while other ponds were used as a watering source for the course. Some of the ponds were very close to, or adjacent to greens, which are heavily managed, while others were in areas where lower management occurred. These differences in management among the golf courses may explain some of the differences seen in survival between buffered and unbuffered ponds on the golf courses. For example, larval green frog survival in unbuffered ponds (Fig. 1A) was highest in Hueston Woods, which was the least chemically managed course, and lowest in Oxford Country Club, which had severe water level fluctuations with moderate chemical management. Also, on Twin Run both green frogs and cricket frogs experienced similar survival in buffered and unbuffered ponds (Figs. 1A and 1B) most likely because the buffer treatment was compromised with the addition of copper sulfate into the buffered pond. Previous studies have shown that anurans are sensitive to copper and have documented effects on survival at low concentrations (12–23 μg/L) [45]–[47].

We anticipated a reduction in contaminant levels with buffer zones, resulting in reduced nutrient loading (which could affect algal food resources) and increased invertebrate predator abundance as found in Boone et al. [16]. Although invertebrate predator densities were similar between buffered and unbuffered ponds on all three golf courses, we did not collect enough information for a quantitative assessment. Therefore, the differences seen in survival could be due, in part, to invertebrate predators. Additionally, we did not see differences in phytoplankton resources that would explain patterns, but periphyton or detritus may have been the main food resources for tadpoles. A lack of differences in larval survival between buffered and unbuffered ponds on some courses, such as Oxford Country Club (Fig. 1A), could imply the buffer zone was not wide enough to affect contaminant levels or that other factors were more important (i.e., like frequent changes in water depth).

Both species had increased mass either at the end of the study (green frogs) or at metamorphosis (cricket frogs) when reared in ponds with buffer zones than when tadpoles were reared in ponds without buffer zones on some courses (Fig. 2AB). The larger mass was not a result of differences in density between buffered and unbuffered ponds because survival was used as a covariate in the analysis. Greater mass could provide both species with fitness advantages later in life such as shorter time to reach sexual maturity [34], [48] or larger size at first breeding [42], [49]. Also, previous research has found that the juvenile stage is the critical life stage for maintaining populations of some amphibians [50]–[51]. Therefore, the buffer zone may have negative or neutral effects on survival, but if greater mass at the end of the study leads to greater mass at or shorter time to metamorphosis, buffer zones could have positive implications for juveniles and thus for populations. Because cricket frogs experienced increased survival and increased mass at metamorphosis on some golf courses when reared in ponds with buffer zones, we would expect to find more cricket frog populations in ponds with a terrestrial buffer zone.

Differences in phytoplankton do not explain the differences in mass, which suggests that tadpoles are eating periphyton and/or detritus rather than phytoplankton [52]–[53]. We did attempt to measure periphyton but were unable to collect good samples from the enclosures. During the study, copper sulfate was directly applied to the buffered pond on Twin Run, a contaminant known to affect amphibian growth [54]. Copper sulfate application could explain the lack of a buffer response from cricket frogs at Twin Run, as the buffer was unable to filter the contaminant as it was directly applied to the water.

### Impact of Buffer Zone on Terrestrial Life Stage

Amphibians require terrestrial habitat to feed, grow, and overwinter, but terrestrial habitat is often overlooked when managing for amphibians [55]. In our experimental study, juvenile cricket frogs generally preferred unmown grass to mown grass when given a choice (Fig. 3), which suggests the unmown grass provides the cricket frogs with some advantage, most likely increased food resources and escape from desiccation (both of which we documented), over the mown grass. We may not have been able to detect a significant preference for the unmown grass because it was more difficult to relocate animals in the unmown grass than in the mown grass, as indicated by the higher detection of adult cricket frogs in mown grass than in unmown grass. Therefore, those animals that we did not recover were probably in the unmown grass leading to an incidental bias toward capturing the frogs in the mown grass. However, there was a strong trend toward cricket frogs preferring unmown habitat vs. mown habitat. Birchfield and Deters [56] found that adult green frogs traveled along the mow line between mown and taller, unmown grass and hypothesized the animals would hop into unmown grass if a person walked near, suggesting that the animal was avoiding potential danger and seeking refuge in unmown grass. In the present study, the unmown grass was more humid and had more insects than the mown grass, indicating that the unmown grass would likely provide greater opportunities to forage and prevent desiccation than the mown grass.

Although in our experimental study we found that cricket frogs had greater probability of choosing unmown grass habitat, we did not find an effect of buffer zone on golf courses because no individuals were recovered. We could have failed to recover cricket frogs for a number of reasons. Released cricket frogs may have perished, dispersed, or we may have failed to detect them. Cricket frogs in natural populations experience very high mortality prior to overwintering, 50–97% of the population in some places [57]–[58]. Cricket frog mortality could have been a result of predation as cricket frogs have many predators, such as larger frogs, birds, fish, snakes, and mammals [59], which may have been present on the golf courses. In fact Scott et al. [15] found that most amphibians on golf courses were green frogs and bullfrogs, which are known to prey on smaller amphibians, such as cricket frogs. Also, it is likely that our sample size was too low to recover animals, especially at sites where fewer than 50 individuals were released. However, in a previous mark-recapture study with anurans, Waddle et al. [60] marked a minimum of 80 treefrogs and recaptured 61% of those individuals during the next several months. Finally, toe-clipping could have reduced the return rate of frogs the following spring; however we should have located at least 50% of the animals taking the number of toes removed into account [61]. Therefore, it is more likely that we were unable to locate any marked animals at any of our sites because they dispersed to other sites or died.

### Conclusion

Larvae of both Blanchard's cricket frogs and green frogs were able to survive in golf course ponds and buffer zones appeared to benefit cricket frogs on some golf courses. Also, juvenile cricket frogs generally preferred unmown grass over mown grass, which may indicate that unmown grass will be important for maintaining cricket frog populations on green spaces like golf courses; however, it is not clear how much buffer zone is needed. Juvenile survival has been implicated as the most critical life stage for maintaining viable populations of other species of amphibians [50]–[51] because fecundity is relatively high, allowing for tolerance of increased larval mortality. If conservation efforts are geared to the more critical life stage of juvenile survival, rather than larval survival, the buffer zone may be important in providing terrestrial habitat regardless of the effects seen during the larval stage.

Our study provides further evidence that larval requirements can be met by many managed wetlands, but the impact of changes in the terrestrial habitat on amphibians is still not well understood. We did find some indication that unmown grass could provide suitable habitat based on cricket frog preference for this habitat. Future work should focus on whether or not amphibians can persist in the terrestrial environment on golf courses and the minimum buffer zone necessary to sustain populations as well as if golf courses are acting as population sinks rather than sustaining populations. Many golf course superintendents are not able to leave a buffer zone around the entire perimeter of a pond. Therefore, determining the amount of buffer zone needed around the perimeter of the pond would be useful. This would provide golf course managers with more detailed guidelines on how to implement buffer zones on their golf courses. Also, studies evaluating if there is an optimum height for grasses to filter contaminants and also provide essential habitat for amphibians would be useful. Evaluating the effects of buffer zones on other grassland species of amphibian and wildlife would also provide more evidence to support the need for buffer zones on golf courses. Golf courses could serve as a model for other managed green spaces and buffer zones could be implemented on many types of aquatic sites. This strategy could provide site managers with an opportunity to reduce environmental impacts and also slow or stop the decline of threatened species like the Blanchard's cricket frog and provide habitat for other native grassland species.
